# Cytokine Profile of Mouse Vaginal and Uterus Lymphocytes at Estrus and Diestrus

**DOI:** 10.1080/17402520500141010

**Published:** 2005-06

**Authors:** Maria C. Lopez, Margaret A. Stanley

**Affiliations:** Department of Pathology University of Cambridge CB2 1QP Cambridge UK

## Abstract

It is known that sex hormones regulate IgA and IgG levels in the female reproductive tract. Moreover, antigen presentation by uterine and vaginal epithelial cells is also under strict hormonal control. The effect of the estrous cycle on cytokine secretion by vaginal and uterine lymphoid cells has been examined in mice using simultaneous staining for cytoplasmic cytokines and surface markers after *ex vivo* culture with PMA/ionomycin in the presence of Brefeldin A, and flow cytometry analysis. Two different mice strains, BALB/c and C57BL/6 mice, were used. The most relevant finding was the increase in the proportion of vaginal cells secreting IFN-γ at diestrus in both strains of mice. Other cytokines (IL-2 and IL-4) as well as some T cell subsets seemed to be modified in a strain dependent fashion. Data also suggest that NK cells are at least partially responsible for IFN-γ secretion. Our data indicate that vaginal and uterus lymphoid cells isolated at diestrus were *in vivo* activated to secrete cytokines after *ex vivo* culture. IFN-γ seems to be the key cytokine, since it increases in both strains of mice.


*Clinical & Developmental Immunology*, June 2005; 12(2): 159–164

Taylor & Francis

Taylor & Francis Group

### Cytokine profile of mouse vaginal and uterus lymphocytes at estrus and diestrus

MARIA C. LOPEZ, & MARGARET A. STANLEY

*Department of Pathology, University of Cambridge, Cambridge CB2 1QP, UK*

**Abstract**

It is known that sex hormones regulate IgA and IgG levels in the female reproductive tract. Moreover, antigen presentation by uterine and vaginal epithelial cells is also under strict hormonal control. The effect of the estrous cycle on cytokine secretion by vaginal and uterine lymphoid cells has been examined in mice using simultaneous staining for cytoplasmic cytokines and surface markers after *ex vivo* culture with PMA/ionomycin in the presence of Brefeldin A, and flow cytometry analysis. Two different mice strains, BALB/c and C57BL/6 mice, were used. The most relevant finding was the increase in the proportion of vaginal cells secreting IFN-γ at diestrus in both strains of mice. Other cytokines (IL-2 and IL-4) as well as some T cell subsets seemed to be modified in a strain dependent fashion. Data also suggest that NK cells are at least partially responsible for IFN-γ secretion. Our data indicate that vaginal and uterus lymphoid cells isolated at diestrus were *in vivo* activated to secrete cytokines after *ex vivo* culture. IFN-γ seems to be the key cytokine, since it increases in both strains of mice.

**Keywords:** *Estrous cycle, vaginal lymphocytes, uterine lymphocytes, cytoplasmic cytokines, NK cells, flow cytometry*

### Introduction

Endocrine control of the estrous cycle is mainly exerted by two steroid hormones: estradiol and progesterone. The cyclic changes are induced on secretory immunity by different hormonal levels (Wira et al. 1999). In rodents, it has been shown that the endocrine changes that take place during the estrous cycle regulate the levels of IgA, IgG and polymeric IgA receptor in the uterine and cervicovaginal secretions (Wira and Stern 1992). Polymeric IgA, its receptor and IgG enter the uterine secretions under the effect of estradiol (Wira and Sandoe 1977), but decline in cervicovaginal secretions (Wira and Sullivan 1985). Similar cyclical changes have been observed in humans (Brandtzaeg 1997).

The presence of immune cells—macrophages, lymphocytes, neutrophils, NK cells, Langerhans cells—in the female reproductive tract and their interactions with epithelial, stromal and endothelial cells have also been shown in humans and rodents (King et al. 1989, Laguens et al. 1990, Nandi and Allison 1991, Parr and Parr 1991, Bjercke and Brandtzaeg 1993, Kaushic et al. 1998). Furthermore, antigen presentation by uterine and vaginal antigen presenting cells in rodents depends on the stage of the estrus cycle (Wira and Rossoll 1995a,b). Uterus and vagina differ while antigen presentation by uterine epithelial cells is high at estrus, the opposite is true for vaginal cells (Wira and Rossoll 1995a).

Altogether, the studies found in the literature suggest a very complex regulation of the immune system in the female reproductive tract that depends on the hormonal status and on the anatomical site. In this study, we examine the effect of hormonal status on cytokine production by lymphoid cells in the uterus and vagina of mice at estrus and diestrus.

### Materials and methods

### *Animals*

BALB/c and C57BL/6 mice 8–12 weeks old were obtained from Charles River UK (Morgate, Kent, UK).

Correspondence: M. C. López, Albany Medical College, Center for Immunology & Microbial Disease, 47 New Scotland Ave. MC-151, Albany, NY 12208, USA. Tel: 1 518 262 6263. Fax: 1 518 262 6161. E-mail: lopezma@mail.amc.edu

ISSN 1740-2522 print/ISSN 1740-2530 online © 2005 Taylor & Francis Group Ltd
DOI: 10.1080/17402520500141010

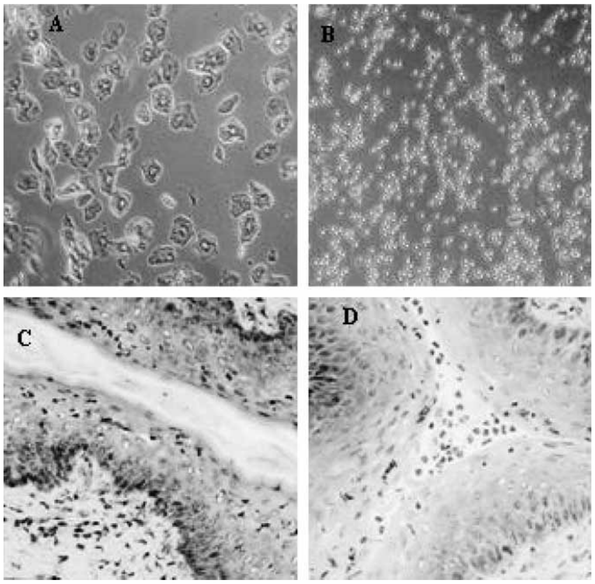

Figure 1. Leukocyte distribution in vagina. Vaginal lavage cells observed under the microscope showed clear differences between estrus (A) and diestrus (B) cell morphology. Hematoxylin and eosin staining of mouse vagina shows leukocytes reaching the basal epithelial layers, and cornified epithelia in the vaginal lumen at estrus (C) while leukocytes fill the vaginal lumen at diestrus (D) (20 × objective).

Vaginal lavage was performed using 50 μl of PBS that were dispensed in the vagina with a plastic tip. Cells were collected and analyzed under a microscope to determine the stage of the estrous cycle. Only mice showing clear middle stages in either estrus or diestrus were chosen, as shown in Figure 1A and B. Estrus was defined by the presence of large numbers of cornified cells and the nearly complete lack of leukocytes in vaginal lavage fluid. Diestrus was characterized by the presence of large numbers of leukocytes and few either rounded or cornified epithelial cells. Five experiments were performed using BALB/c mice, with 12–20 mice at estrus and 11–20 mice at diestrus. Cell yield for vaginas was $(2.14 \pm 0.93) \times 10^5$ at estrus and $(1.87 \pm 0.60) \times 10^5$ at diestrus. Cell yield for uterus was $(2.41 \pm 1.19) \times 10^6$ at estrus and $(2.31 \pm 0.79) \times 10^5$ at diestrus. Four experiments were performed using C57BL/6 mice, with 16–18 mice at estrus and 11–15 mice at diestrus. Cell yield for vaginas was $(1.12 \pm 0.25) \times 10^5$ at estrus and $(1.76 \pm 0.60) \times 10^5$ at diestrus. Cell yield for uterus was $(2.87 \pm 1.17) \times 10^6$ at estrus and $2.72 \times 10^5 \pm 0.75 \times 10^5$ at diestrus. Data are presented as mean ± standard error of 2–4 experiments, since not all markers were studied simultaneously. Data from 2–4 experiments using pooled vaginal or uterine cells collected at estrus or diestrus were compared using the Student's *t* test included in Excel software.

*Lymphocyte isolation and culture*

Mice were sacrificed and the vagina and uterus from each mouse was isolated and pooled according to their stage in the estrous cycle. Single cells suspensions from vaginas were obtained following a procedure described previously, with slight modifications (Nandi and Allison 1991, 1993). Briefly, after cutting the vagina into small pieces with a scalpel blade, all pieces were incubated in Hank's balanced salt solution containing 0.1 mg/ml collagenase/dispase (Sigma, St Louis, MO) and 0.1 mg/ml DNase I (Roche Diagnostics, Lewes, UK) for 1 h in a bacterial shaker at 210 rpm and at 37°C. Uteri were cut into smaller pieces with scissors and were incubated in HBSS containing DNase I in the same conditions used for vaginas. At the end of the incubation period the cell suspensions were first passed through a cell strainer (Falcon 2350, BD Biosciences, San Jose, CA) where large pieces of tissue were pressed with the help of a syringe plunger, and then through nylon/cotton wool columns to eliminate dead cells and debris. Cells were spun down, washed in complete medium (CM) containing RPMI 1640 (Gibco, Carlsbad, CA) supplemented with 10% heat inactivated fetal calf serum (Gibco), 20 mM HEPES, 0.05 mM mercaptoethanol and antibiotics (penicillin, streptomycin), and spun down again. Finally, cells were resuspended in CM and layered on top of Lympholite-M (Cedarlane, Hornby, Ontario, Canada). Gradients were spun down for 20 min at room temperature, at 1900 rpm. Cells were washed in CM and counted to determine viability in the presence of trypan blue. Then cells were ready to incubate in the presence of mitogens to induce cytokine secretion. Mesenteric lymph nodes were isolated from one mouse in each experiment, and prepared by pressing through a cell strainer (Falcon 2340); cells were collected on CM, washed, resuspended in 2 ml and cultured in exactly the same conditions as vaginal and uterine cells.

In order to induce cytokine synthesis and to facilitate its retention within the cytoplasm, a published protocol with slight modifications was followed (Openshaw et al. 1995). Pooled cell suspensions were resuspended in 2 ml, were deposited in a well of a 24 well plate, and stimulated with PMA (50 ng/ml) (Sigma) plus ionomycin (500 ng/ml) (Sigma) for 6 h at 37°C, in the presence of Brefeldin A (Epicentre Technologies, Madison, WI) at a 10 μg/ml dilution. After the incubation period, cells were ready for surface markers and cytoplasmic cytokine determination.

*Flow cytometric simultaneous analysis of surface markers and intracellular cytokines*

The procedure was performed as previously described (López and Stanley 2000). At the end of the incubation period, cells were resuspended in their wells and transferred to a 96 well V-bottom plate to perform the staining. Plates were spun down, vortexed, and 100 μl of PBS/2%BSA/0.01% Na azide, supplemented with Brefeldin A, were added to each well. Cells were fixed with 100 μl of 4% paraformaldehyde (pH 7.4) per well and the plate was incubated on ice for 20 min. Plates were spun down and cells were resuspended in PBS/2%BSA/0.5% saponin/0.01% Na azide, and incubated in the cold for other 20 min, to facilitate pore formation. Then, fluorochrome-conjugated antibodies that recognize mouse cytokines were added. Plates were incubated in the cold room with shaking for 30 min. Then, they were spun down, washed twice with PBS/2%BSA/0.5% saponin, and once with PBS/2%BSA. Antibodies recognizing specific surface markers were added, and plates were incubated with shaking in the cold room for 30 min. Afterwards, plates were spun down and the cells were washed with PBS/2%BSA. Finally, cells were resuspended in 2% paraformaldehyde, stored in darkness at 4°C, until analyzed. Samples were always run within 24 h after the staining was finished. Samples were run in a Facscan (BD Immunocytometry systems, San Jose, CA) and results were analyzed using Cellquest software (BD). All samples were run in the same conditions for forward and side scatter, FL1, FL2 and FL3. Lymphocytes were first gated according to their scatter characteristics. The lymphocyte gate was determined based upon the lymphocyte gate for mesenteric lymph node cells that were always run as controls; but allowing for higher forward and side scatter. In all experiments unstained cells and cells stained separately with each fluorochrome were included to optimize compensation settings. Samples were analyzed for the expression of surface markers and cytoplasmic cytokines. Samples were further analyzed by re-gating on $CD4^+$, $CD8^+$, $\gamma\delta$-$TCR^+$, $CD3^+$, $DX5^+$ cells and the percentage of cells secreting cytokines within the new gate was determined. To calculate the percentage of cells producing cytokines, the cursor was set up to exclude the nonspecific binding produced by the respective directly conjugated isotype control. For NK cells, the percentage of cells double positive for DX5 and IFN-γ within the lymphocyte gate was also calculated.

The following directly conjugated antibodies were used in this study: FITC anti-mouse IL-2, PE anti-mouse IFN-γ, FITC anti-mouse pan-NK cell marker (clone DX5), CyChrome anti-mouse CD3, and FITC rat IgG1 (Pharmingen, San Diego, CA); PE anti-mouse IL-4, FITC anti-mouse IL-10, Tricolor anti-mouse CD4, Tricolor anti-mouse CD8α, Tricolor anti-mouse γδ-TCR, FITC rat IgG2a and PE rat IgG2b (Caltag, Burlingame, CA).

### Results

### *Distribution of leukocytes in vaginas at estrus and diestrus*

Leukocyte distribution in vagina and uterus was examined only at estrus and diestrus where the morphological changes induced by the hormonal changes were most marked. Data presented in Figure 1 show the difference between materials obtained from vaginal lavage at estrus and diestrus and the correlation with hematoxylin eosin staining in paraffin embedded sections. At estrus, the vaginal lumen showed large numbers of cornified epithelial cells, leukocytes migrated within the stromal cell layers reaching the basal epithelia, with intraepithelial lymphocytes within the basal and parabasal epithelial layers (Figure 1A and C). This population of leukocytes included neutrophils, macrophages and also lymphocytes (Figure 1C). At diestrus leukocytes were observed crossing the superficial epithelial cell layers reaching the vaginal lumen (Figure 1D) and were collected in the vaginal lavage (Figure 1B). Some lymphocytes could also be found amongst epithelial cells (Figure 1D). Lymphocytes were dominant in both stroma and epithelium at diestrus.

### *Distribution of T and NK cells and cytokine secreting cells after short term culture of vaginal and uterine cells isolated at estrus and diestrus*

Lymphocytes from vaginas and uterus were isolated as mentioned above and the cells were cultured and stained to simultaneously analyze surface markers and intracellular cytokines. Data obtained from vaginal cells studying the distribution of T and NK cell markers after culture, showed a higher proportion of $CD4^+$ cells at diestrus in C57BL/6 mice ($p < 0.004$) (Figure 2A). No changes were observed when the percentage of cells expressing T and NK cell markers in the uterus was analyzed in these mouse strains (Figure 2B).

Vaginal and uterine cells were stimulated *ex vivo* for 6 h with PMA and ionomicyn in the presence of Brefeldin A to favor the intracellular accumulation of cytokines. This assay allows the study of cells that have been differentiated *in vivo* to produce a relevant mRNA that under these culture conditions will be translated into protein. When vaginal cells secreting different cytokines were analyzed, a higher proportion of cells secreting IFN-γ was demonstrated at diestrus in both strains of mice (Figure 3A). Moreover, an increase in the proportion of cells secreting either IL-2 or IL-4 at diestrus when compared with estrus was also demonstrated in BALB/c ($p < 0.03$) and C57BL/6 ($p < 0.001$) mice, respectively. Conversely, no statistically significant changes were observed when uterine cells were analyzed for cytokine secretion (Figure 3B).

### *$CD4^+$ cells secreting IFN-γ, IL-2, IL-4 and IL-10 and NK cells secreting IFN-γ in vaginas at estrus and diestrus*

Data presented for vaginal cells was re-analyzed by gating on a specific T or NK cell marker and studying

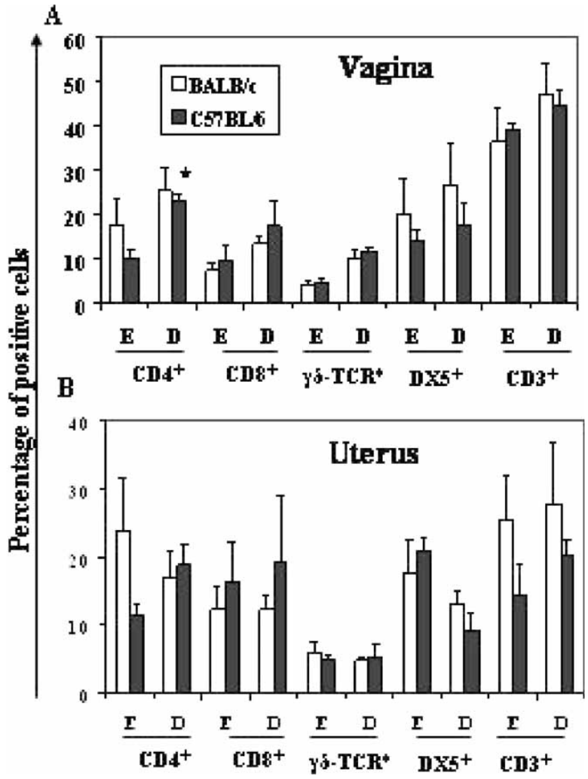

Figure 2. Vaginal (A) and uterine (B) T and NK cells at estrus and diestrus after *ex vivo* culture for 6 h in the presence of PMA/ION and Brefeldin A. (estrus vs. diestrus: $^{\star}p < 0.004$).

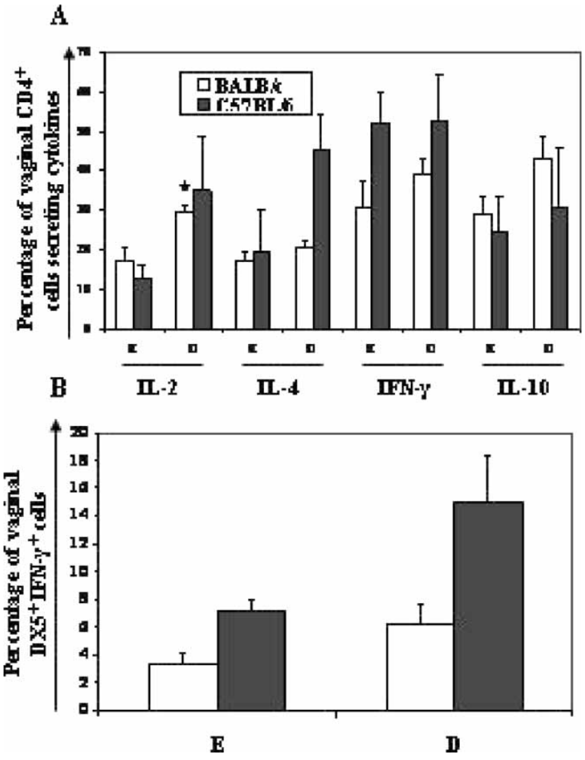

Figure 4. Vaginal CD4$^{+}$ cells secreting IL-2, IL-4, IFN-γ and IL-10 (A) and DX5$^{+}$ IFN-γ$^{+}$ cells (B) at estrus and diestrus in the same conditions mentioned in Figure 2 (estrus vs. diestrus: $^{*}p < 0.02$).

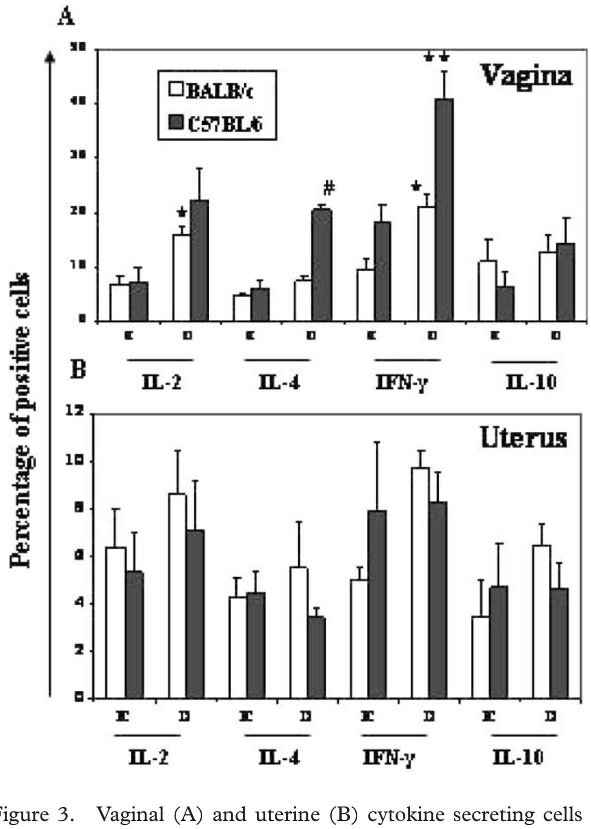

Figure 3. Vaginal (A) and uterine (B) cytokine secreting cells at estrus and diestrus after *ex vivo* culture as mentioned in Figure 2 (estrus vs. diestrus: $^{\star}p < 0.03$; $^{\#}p < 0.001$; $^{\star\star}p < 0.04$).

the percentage of cells secreting cytokines. By using this procedure it was established that the percentage of CD4$^{+}$ cells producing IL-2 was higher at diestrus than at estrus in the vaginas of BALB/c mice (Figure 4A). No further statistical differences were found.

To further understand the role of NK cells in IFN-γ production, data were also analyzed as the percentage of DX5 IFN-γ double positive cells. Although there was a tendency to increase percentages at diestrus the trend was not statistically significant (Figure 4B).

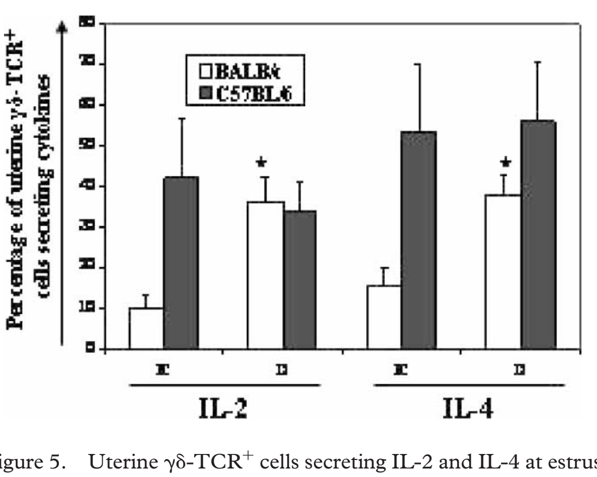

Figure 5. Uterine γδ-TCR$^{+}$ cells secreting IL-2 and IL-4 at estrus and diestrus in the same conditions mentioned in Figure 2. (estrus vs. diestrus: $^{*}p < 0.02$).

*γδ-TCR$^+$ cells secreting IL-2 and IL-4 in uterus at estrus and diestrus*

Data obtained for uterine cells was re-analyzed by gating on a T or NK cell marker and studying the percentage of cells secreting cytokines. Thence, it was possible to demonstrate a higher percentage of γδ-TCR$^+$ cells producing IL-2 and IL-4 at diestrus in the uterus of BALB/c mice (**Figure** 5). No further differences could be demonstrated.

### Discussion

It has been clearly shown that IgA and IgG levels in uterine secretions change in accordance to the stage of the estrous cycle, reaching higher levels at the time of ovulation (Wira and Sandoe 1977). These early findings led to a series of investigations trying to understand the effects of estrogen and progesterone on secretory immune system regulation and antigen presentation in the female reproductive tract (Wira et al. 1999). Other studies have shown that sex hormones can also modulate lymphocyte function at sites different from the female genital tract. Diminished graft versus host capacity of donor uterine draining lymph node cells was observed at estrus (Bonaparte et al. 1986) and was associated with a decrease in the percentage and absolute number of CD8 cells (Colombo et al. 1988). Moreover, studies performed in humans using peripheral blood mononuclear cells indicated that the secretion of TNF-α and IL-4 was directly correlated with the levels of estrogens in pre menopausal women (Schwarz et al. 2000, Verthelyi and Klinman 2000). Nevertheless, there is a paucity of studies analyzing genital tract associated lymphoid cells and their cytokine secretion profile.

In this report, flow cytometry analysis of isolated mouse vaginal and uterine lymphoid T and NK cells was used to study cell distribution and cytokine secretion profile at estrus and diestrus. A higher proportion of CD4$^+$ cells was only found in C57BL/6 mice vagina, at diestrus. This increase could be either due to an increase in recruitment in response to hormonal induced chemokine release or proliferation *in situ* (Sonoda et al. 1998). Previous studies have indicated a low level of circulation between the periphery and the vaginal mucosa (Fidel et al. 1997). Although it has been shown that vaginal T cells were able to proliferate *in situ* in response to systemic T cell stimuli, they are considered thymic derived or thymic factor dependent due to their paucity in nude mice (Ibraghimov et al. 1995).

After ex vivo stimulating vaginal and uterine cells that had already been differentiated *in vivo* to produce cytokines, the percentage of vaginal cells secreting IFN-γ was found to be increased at diestrus, in both strains of mice. Furthermore, there was an increase in the percentage of cells secreting IL-2 in BALB/c mice and IL-4 in C57BL/6 mice. Lymphocyte activation associated with increased cytokine secretion at diestrus may be necessary in case of infection, since the natural barrier formed by several layers of cornified epithelial cells disappears at the end of the estrus stage. Susceptibility to Herpes Simplex virus-2 and *Chlamydia trachomatis* vaginal infections in mice have been shown to be associated with either diestrus or administration of progesterone (Parr et al. 1994, Gallichan and Rosenthal 1996, Kaushic et al. 2000). However, successful infection with *Candida albicans* was associated with high estrogen levels (Fidel et al. 2000). Interestingly, when the percentage of vaginal DX5$^+$ IFN-γ$^+$ was analyzed, a tendency to increase values at diestrus was observed in both strains of mice, nonetheless the difference was not statistically significant. Nevertheless, the finding that NK cells were located in mouse vagina and secreting IFN-γ reinforces previous findings that indicated that NK cell secretion of IFN-γ was crucial in the early stages of Herpes Simplex virus-2 resolution (Milligan and Bernstein 1997, Ashkar and Rosenthal 2003).

Changes in cytokine secretion profile were not observed when uterine lymphocytes were analyzed. Nevertheless, when flow cytometry data were re-analyzed by gating on different T-cell markers and studying their specific cytokine secretion profile, an increase in the percentage of γδ-TCR$^+$ cells secreting IL-2 and IL-4 was observed in BALB/c mice. These data suggest that hormonal levels could modify the activation of specific lymphocyte subsets in a strain dependent fashion.

Finally, our results point to a complex interaction between sex hormone levels, the mucosal immune system, specific pathogens and genetic background at the level of the female reproductive tract. In conclusion, our data indicate that vaginal and uterus lymphoid cells isolated at diestrus had been differentially activated *in vivo* to secrete cytokines after *ex vivo* re-stimulation. IFN-γ secreted by NK cells seems to be the key cytokine, since it was found increased in both strains of mice.

### References

Ashkar AA, Rosenthal KL. 2003. Interleukin-15 and natural killer and NKT cells play a critical role in innate protection against genital herpes simplex virus type 2 infection. J Virol 77:10168–10171.

Bjercke S, Brandtzaeg P. 1993. Glandular distribution of immunoglobulins, J chain, secretory component, and HLA-DR in the human endometrium throughout the menstrual cycle. Hum Reprod 8:1420–1425.

Bonaparte YP, Colombo LL, Klein S, D'Elia I, Diament M. 1986. Graft vs Host reaction induced by paraaortic lymph nodes during estrous and diestrous stages. Am J Reprod Immunol 12:45–47.

Brandtzaeg P. 1997. Mucosal immunity in the female genital tract. J Reprod Immunol 36:23–50.

Colombo LL, Lopez MC, Herkovits J, Roux ME. 1988. T and B lymphocyte populations in uterus draining lymph nodes at estrus and diestrus in Wistar rats. Am J Reprod Immunol Microbiol 16:143–145.

Fidel Jr., PL, Luo W, Chabain J, Wolf NA, Van Buren E. 1997. Use of cellular depletion analysis to examine circulation of immune effector function between the vagina and the periphery. Infect Immun 65:3939–3943.

Fidel Jr., PL, Cutright J, Steele C. 2000. Effects of reproductive hormones on experimental vaginal candidiasis. Infect Immun 68:651–657.

Gallichan WS, Rosenthal KL. 1996. Effects of the estrous cycle on local humoral immune responses and protection of intranasally immunized female mice against herpes simplex virus type 2 infection in the genital tract. Virology 224:487–497.

Ibraghimov AR, Sacco RE, Sandor M, Iakoubov LZ, Lynch RG. 1995. Resident $CD4^+$ $\alpha\beta$ T cells of the murine female tract: A phenotypically distinct T cell lineage that rapidly proliferates in response to systemic T cell stimuli. Int Immunol 7:1763–1769.

Kaushic C, Frauendorf E, Rossoll RM, Richardson JM, Wira CR. 1998. Influence of the estrous cycle on the presence and distribution of immune cells in the rat reproductive tract. Am J Reprod Immunol 39:209–216.

Kaushic C, Zhou F, Murdin AD, Wira CR. 2000. Effects of estradiol and progesterone on susceptibility and early immune responses to *Chlamydia trachomatis* infection in the female reproductive tract. Infect Immun 68:4207–4216.

King A, Wellings V, Loke YW. 1989. Immunocytochemical characterization of unusual large granular lymphocytes in human endometrium throughout the menstrual cycle. Hum Immunol 24:195–205.

Laguens G, Goni JJ, Laguens M, Goni JM, Laguens R. 1990. Demonstration and characterization of HLA-DR positive cells in the stroma of human endometrium. J Reprod Immunol 18:179–186.

López MC, Stanley MA. 2000. Cytokine profile of draining lymph node lymphocytes in mice grafted with syngeneic keratinocytes expressing human papillomavirus type 16 E7 protein. J Gen Virol 81:1175–1182.

Milligan GN, Bernstein DL. 1997. Interferon-gamma enhances resolution of herpes simplex virus type 2 infection of the murine genital tract. Virology 229:259–268.

Nandi D, Allison JP. 1991. Phenotypic analysis and $\gamma\delta$-T cell receptor repertoire of murine T cells associated with the vaginal epithelium. J Immunol 147:1773–1778.

Nandi D, Allison JP. 1993. Characterization of neutrophils and T lymphocytes associated with the murine vaginal epithelium. Reg Immunol 5:332–338.

Openshaw PJM, Murphy EE, Hosken NA, Maino V, Dvis K, Murphy K, O'Garra A. 1995. Heterogeneity of intracellular cytokine synthesis at the single-cell level in polarized T helper 1 and T helper 2 populations. J Exp Med 182:1357–1367.

Parr MB, Parr EL. 1991. Langerhans cells and T lymphocyte subsets in the murine vagina and cervix. Biol Reprod 44:491–498.

Parr MB, Kepple L, McDermott MR, Drew MD, Bozzola JJ, Parr EL. 1994. A mouse model for studies of mucosal immunity to vaginal infection by herpes simplex virus type 2. Lab Invest 70:369–380.

Schwarz E, Schäfer C, Bode JC, Bode C. 2000. Influence of the menstrual cycle on the LPS-induced cytokine response of monocytes. Cytokine 12:413–416.

Sonoda Y, Mukaida N, Wang J-B, Shimada-Hiratsuka M, Naito M, Kasahara T, Harada A, Inoue M, Matsushima K. 1998. Physiologic regulation of postovulatory neutrophil migration into vagina in mice by a C-X-C chemokine(s). J Immunol 160:6159–6165.

Verthelyi D, Klinman DM. 2000. Sex hormone levels correlate with the activity of cytokine-secreting cells *in vivo*. Immunology 100:384–390.

Wira CR, Rossoll RM. 1995a. Antigen presenting cells in the female reproductive tract: Influence of sex hormones on antigen presentation in the vagina. Immunology 84:505–508.

Wira CR, Rossoll RM. 1995b. Antigen-presenting cells in the female reproductive tract: Influence of the estrous cycle on antigen presentation by uterine epithelial and stromal cells. Endocrinology 136:4526–4534.

Wira CR, Sandoe CP. 1977. Sex steroid hormone regulation of IgA and IgG in rat uterine secretions. Nature 268:534–536.

Wira CR, Sullivan DA. 1985. Estradiol and progesterone regulation of IgA, IgG and secretory component in cervico-vaginal secretions of the rat. Biol Reprod 32:90–95.

Wira CR, Stern J. 1992. Endocrine regulation of the mucosal immune system in the female reproductive tract: Control of IgA, IgG, and secretory component during the reproductive cycle, at implantation and throughout pregnancy. In: Pasqualini R, Scholler R, editors. Hormones and fetal pathophysiology. New York: Marcel Dekker. pp 343–368.

Wira CR, Kaushic C, Richardson J. 1999. Role of sex hormones and cytokines in regulating the mucosal immune system in the female reproductive tract. In: Ogra PL, Mestecky J, Lamm ME, Strober W, Bienenstock J, McGhee JR, editors. Mucosal immunology. San Diego: Academic Press. pp 1449–1461.

